# Antibacterial Properties of PMMA/ZnO(NanoAg) Coatings for Dental Implant Abutments

**DOI:** 10.3390/ma18020382

**Published:** 2025-01-15

**Authors:** Ana Maria Gianina Rehner (Costache), Dana-Ionela Tudorache, Alexandra Cătălina Bîrcă, Adrian Ionuț Nicoară, Adelina-Gabriela Niculescu, Alina Maria Holban, Ariana Hudiță, Florentina Cornelia Bîclesanu, Paul Cătălin Balaure, Anna Maria Pangică, Alexandru Mihai Grumezescu, George-Alexandru Croitoru

**Affiliations:** 1 Faculty of Medicine, Titu Maiorescu University, 031593 Bucharest, Romania; rehner.ana@gmail.com (A.M.G.R.); corneliabicle@yahoo.com (F.C.B.); anna-maria.pangica@prof.utm.ro (A.M.P.); 2 Faculty of Chemical Engineering and Biotechnologies, University Politehnica of Bucharest, Gh. Polizu Str. 1–7, 060042 Bucharest, Romania; dana.tudorache@upb.ro (D.-I.T.); ada_birca@yahoo.com (A.C.B.); adrian.nicoara@upb.ro (A.I.N.); adelina.niculescu@upb.ro (A.-G.N.); paul.balaure@upb.ro (P.C.B.); 3 Research Institute of the University of Bucharest—ICUB, University of Bucharest, 050657 Bucharest, Romania; arianahudita@yahoo.com; 4 Faculty of Biology, University of Bucharest, Aleea Portocalelor 1–3, Sector 5, 030018 Bucharest, Romania; alina_m_h@yahoo.com; 5 Faculty of Dental Medicine, Carol Davila University of Medicine and Pharmacy, 8 Eroii Sanitari Street, 050474 Bucharest, Romania; alex.croitoru@umfcd.ro

**Keywords:** antimicrobial coatings, abutment infection, hydrothermal synthesis, spin coating method, PMMA—zinc oxide—silver nanoparticles composite

## Abstract

Infections continue to pose significant challenges in dentistry, necessitating the development of innovative solutions that can effectively address these issues. This study focuses on creating coatings made from polymethyl methacrylate (PMMA) enriched with zinc oxide–silver composite nanoparticles, layered to Ti6Al4V–titanium alloy substrates. The application of these materials aims to create a solution for the abutments utilized in complete dental implant systems, representing the area most susceptible to bacterial infections. The nanoparticles were synthesized using a hydrothermal method, optimized through specific temperature and pressure parameters to achieve effective morphologies and sizes that enhance antibacterial efficacy. The layers were applied to the titanium substrate using the spin coating technique, chosen for its advantages and compatibility with the materials involved. Comprehensive analyses were conducted on the antimicrobial powders, including X-ray diffraction, Fourier transform infrared spectroscopy, scanning electron microscopy, and energy-dispersive X-ray spectroscopy. Furthermore, the PMMA-based coatings incorporating antimicrobial nanoparticles were evaluated to ensure uniformity and homogeneity across the titanium alloy surface by IR mapping and SBF immersion–SEM analysis. The antimicrobial activity of the samples was demonstrated with impressive results against *Staphylococcus aureus*, *Pseudomonas aeruginosa*, and *Candida albicans*, as assessed through biofilm modulation studies. The biocompatibility of the samples was validated through in vitro cell-based assays, which demonstrated excellent compatibility between PMMA-based coatings and human preosteoblasts, confirming their potential suitability for future use in dental implants.

## 1. Introduction

Prosthetic suprastructures, including crowns and both fixed and removable prostheses, rely on a foundational element known as a dental abutment, which continues with the implant. This implant serves as an artificial dental root, providing the necessary stability and support the prosthetic devices to maintain their position and functionality. Dental implants have been utilized for many years, and it is remarkable that the titanium-based materials used in the early pioneering efforts continue to be validated, supported, and widely used today [1,2,3,4]. However, there is a growing interest in the study of implant abutments, which serve as the critical interface connecting the implant body to the prosthetic suprastructure. Given that abutments serve as the crucial link between the implant and the crown, they are designed to accommodate both contact points effectively. Each abutment comprises four essential components: an implant–abutment connection, a flat shoulder, a retentive attachment segment, and a transgingival segment. Titanium-based abutments exhibit specific characteristics, such as surface irregularities along the retentive attachment segment, which enhance the resistance of crown fixation. Additionally, they feature anti-rotation mechanisms at the connection point with the endosseous implant, further ensuring stability [5,6,7]. The primary material associated with implant and abutments is titanium, along with titanium alloys, which have proven to be effective and reliable throughout the decades. Among the key properties that position titanium and titanium alloy implants as top choices for specialists are their excellent biocompatibility and remarkable osseointegration ability [8,9,10].

On the other hand, a significant concern is the potential for the body to react negatively to the entire implant system, leading to inflammatory responses in the surrounding tissue. This issue is classified with peri-implant diseases, which are primarily of two types: peri-implant mucositis and peri-implantitis. These conditions are characterized by polymicrobial inflammation that adversely affects the surrounding tissues, and without prompt and appropriate treatment, they can result in the loss of both the teeth and the associated implants [11].

Peri-implant mucositis refers to the inflammatory response of the soft tissues around the implant. This condition is reversible and is typically identified by symptoms such as swelling, redness, and bleeding, without any damage to the hard tissues surrounding the implant [12,13,14].

In contrast, peri-implantitis represents an irreversible infection characterized by a cascade of detrimental effects, including the loss of bone tissue around the implant, the presence of infection exudate, and the formation of gingival pockets. Diagnosis is made based on clinical observations of swelling, redness, gingival recession, purulent exudate, and significant bone loss as evidenced by radiological imaging [15,16,17].

Bacterial infection involves intricate interactions among bacteria, the surface of the implant system, and the body’s immune response. Initially, bacteria adhere to the surface of the implant material, entering a sessile state where they form microcolonies and protective biofilms that enhance their survival within the host. The body responds by activating its immune system, resulting in a prolonged inflammatory response. Over time, this inflammation leads to the diagnosis of peri-implant mucositis, characterized by altered microcirculation and an increase in leukocyte count. The progression to peri-implantitis is marked by a significant accumulation of inflammatory cells in the affected area and the extension of inflammation into the surrounding bone tissue. This stage also triggers bone resorption, driven by the increased activity of osteoclasts on the bone surface [18,19,20]. The primary factor contributing to the development of peri-implant diseases is the development of biofilms [13].

Understanding the causes of peri-implant diseases and the processes involved in their interaction with implant systems is crucial for designing, developing, and implementing effective prevention methods and establishing appropriate therapeutic strategies [21].

Due to the contextual understanding, research suggests that a promising alternative for addressing this issue involves modifying the surfaces of implant materials with protective coatings that possess antibacterial properties, such as nanostructured coatings designed for drug release [22,23]. Thus, the design of the implant surface must create a secure environment for the body while effectively preventing bacterial colonization from the oral cavity, ideally mimicking a sterilization effect. Furthermore, surface modifications should not only incorporate inherent antimicrobial properties but also actively promote optimal osseointegration [24,25].

There is an undeniable need to develop solutions that minimize the risk of post-implantation infections. Given our current scientific advancements, we can turn to smart materials as a viable option. These materials leverage nanotechnology to enhance surface science, incorporating nanoparticles designed for the localized delivery and controlled release of antimicrobial agents. Additionally, they can promote bone regeneration in the specific anatomical areas surrounding the implant [26].

In this study, we explored a novel coating material for abutment surfaces utilizing polymethyl methacrylate (PMMA) as the polymer matrix, enhanced with zinc oxide (ZnO) and silver (Ag) nanoparticles. Extensive research has demonstrated zinc oxide and silver nanoparticles’ remarkable effectiveness in inhibiting biofilm formation by targeting bacterial cells [27,28,29,30]. By uniformly dispersing these nanoparticles within a stable polymer matrix, we can create a durable and efficient surface treatment that promotes long-term antimicrobial activity. PMMA is widely used in biomedicine, particularly in dentistry, due to its advantageous properties, including ease of handling, aesthetic appeal, affordability, low density, and favorable mechanical characteristics [31,32,33,34]. This innovative material design not only offers a feasible solution for industry applications but also provides significant added value for patients at risk of infections.

## 2. Materials and Methods

### 2.1. Materials

All reagents used for nanoparticle synthesis and film preparation were analytical grade and obtained from Sigma-Aldrich/Merck (Darmstadt, Germany), without further purification. The titanium alloy plates (Ti6Al4V) used in this study were Grade 4 ELI and were purchased from Bibus Metals AG (Essen, Germany).

#### 2.1.1. Synthesis of Zinc Oxide Nanoparticles

The synthesis of zinc oxide (ZnO) nanoparticles utilized zinc nitrate hexahydrate (Zn(NO_3_)_2_·6H_2_O) and sodium hydroxide (NaOH) as the primary reactants. Methanol (CH_4_O) was used as a solvent, and ultrapure water was employed to maintain solution purity throughout the process.

#### 2.1.2. Synthesis of Zinc Oxide-Silver Nanoparticles

For the preparation of zinc oxide–silver (ZnO-Ag) nanoparticles, the following materials were used: zinc nitrate hexahydrate (Zn(NO_3_)_2_·6H_2_O), sodium hydroxide (NaOH), and silver nitrate (AgNO_3_). D-glucose (C_6_H_12_O_6_) served as the reducing agent, and the solvent system consisted of methanol (CH_4_O) and ultrapure water.

#### 2.1.3. Preparation of Antimicrobial PMMA Films on Titanium Alloy Substrates

To create polymethyl methacrylate (PMMA) films, titanium alloy plates (Ti6Al4V) were utilized as the substrate. PMMA ((C_5_O_2_H_8_)_n_) was dissolved in acetone (C_3_H_6_O) to form a uniform solution suitable for spin-coating onto the titanium alloy. The addition of ZnO and ZnO-Ag nanoparticles create coating materials for the antimicrobial films.

### 2.2. Methods

#### 2.2.1. Synthesis Methods for Nanoparticles

##### Synthesis of Zinc Oxide (ZnO) Nanoparticles

Zinc oxide (ZnO) nanoparticles were synthesized using the hydrothermal method. This process began with the preparation of two distinct solutions, which were subsequently combined and subjected to elevated temperatures and pressure. Specifically, a zinc nitrate hexahydrate solution was prepared at a concentration of 0.06 M, with a total volume of 200 mL of methanol. Simultaneously, a sodium hydroxide solution was prepared at a concentration of 0.5 M in 100 mL of ultrapure water. During continuous stirring, the sodium hydroxide solution was added dropwise to the zinc nitrate precursor solution. A noticeable color change occurred, signaling the formation of ZnO nanoparticles. The resulting mixture was then transferred to a Teflon-lined vessel and heated to 160 °C at a pressure of 7 bar for 20 min, allowing 5 min for the target temperature to be reached and 15 min for the reaction to proceed. The final product was subsequently centrifuged three times at 6000 rpm for 10 min per cycle to remove any residual impurities.

##### Synthesis of Zinc Oxide-Silver (ZnO-Ag) Nanoparticles

The synthesis of ZnO-Ag nanoparticles employs a similar approach to that used for ZnO nanoparticles, with a few key differences. In this method, 20 mL of silver nitrate solution at a concentration of 0.02 M is added to the zinc precursor solution. Additionally, a 0.25 M sodium hydroxide solution with a total volume of 40 mL is introduced to the precipitation solution to ensure proper homogenization. To further enhance the reaction, 100 mg of D-glucose is also added to the mixture. Subsequently, the solution was processed using the hydrothermal synthesis technique, maintaining the same parameters as in the synthesis of zinc oxide nanoparticles. Upon completion of the synthesis, the precipitate was subjected to several washings and centrifugations to remove any unwanted residues.

#### 2.2.2. Synthesis Methods for Film Preparation

##### Preparation of Polymethyl Methacrylate (PMMA) Films on Ti6Al4V Substrate

To obtain PMMA films on a titanium alloy substrate, a 0.2 mM solution of PMMA in acetone was prepared. Afterwards, the solution was introduced into a syringe specific to the spin-coating equipment and was placed in the central drip spot in the cover of the deposition equipment. The substrate of Ti6Al4V had dimensions of 1/1 cm and was placed above the vacuum hole; successive drops were applied to the surface of each deposited layer, with a total of five layers. The equipment parameters were set at 2000 revolutions per minute and each layer was obtained in a time period of 20 s. A Laurell Technologies Corporation WS-650 series (Lansdale, PA, USA) spin coater was used.

##### Preparation of PMMA Films Containing ZnO and ZnO-Ag Nanoparticles on Ti6Al4V Substrate

The preparation process for PMMA films incorporating ZnO and Zno-Ag nanoparticles closely mirrors that of the control PMMA films. To achieve a uniform distribution of the nanoparticles, 100 mg of ZnO and ZnO-Ag, respectively, powder was homogenized in 5 mL of PMMA solution. This mixture was then utilized to produce PMMA ZnO coatings and PMMA ZnO-Ag coatings on the titanium alloy substrate.

### 2.3. Characterization Techniques

#### 2.3.1. X-Ray Diffraction

The phase identification and crystallinity properties of the samples were analyzed using X-ray diffraction (XRD) techniques. A PANalytical Empyrean X-ray diffractometer (PANalytical, Almelo, The Netherlands) equipped with a hybrid monochromator on the incident side and a parallel plate collimator mounted on the PIXcel 3D detector was employed for this analysis. Measurements were conducted at room temperature using Grazing Incidence X-ray Diffraction, with 2θ Bragg angle intervals ranging from 20° to 80°. The analysis utilized Cu Kα radiation (λ = 1.5406 Å) at a current of 40 mA and a voltage of 45 kV.

#### 2.3.2. Fourier Transform Infrared Spectroscopy

To identify functional compositional groups, Fourier Transform Infrared (FTIR) analysis was conducted. The spectra were collected using a Thermo iN10-MX FTIR spectrometer with a ZnSe crystal, sourced from Thermo Fisher Scientific in Waltham, MA, USA. The measurements covered the range from 4000 to 400 cm^−1^.

#### 2.3.3. Scanning Electron Microscopy

Morphostructural information regarding the organization of nanoparticles, both individually and as a coating, as well as their sizes, was investigated using a scanning electron microscope (SEM), model Inspect F50, acquired from Thermo Fisher—FEI (Eindhoven, The Netherlands). The images were captured utilizing the secondary electron module at an energy of 30 keV and a spot size of 3.5. Backscatter detection mode was also employed to enhance contrast and reveal compositional differences within the samples. Additionally, elemental composition data were obtained through Energy Dispersive Spectroscopy (EDS), which is integrated into the scanning electron microscope system.

#### 2.3.4. Infrared Microscopy

Infrared analyses of samples prepared using the spin coating method were conducted in reflection mode using a Nicolet™ iN10 MX FT-IR microscope (Thermo Fisher Scientific, Waltham, MA, USA), which is equipped with a liquid nitrogen-cooled MCT detector. For each sample, 32 individual scans were collected across a spectral range of 4000 to 700 cm^™1^, with a resolution of 4 cm^™1^. The collected data were then processed and converted into absorbance spectra using the OMNICPicta™ software package (version 1, Thermo Fisher Scientific, Waltham, MA, USA).

#### 2.3.5. Antimicrobial Assay—Biofilm Development

The microorganisms used for this evaluation were reference strains obtained from the American Type Culture Collection (ATCC): *Staphylococcus aureus* ATCC 6538, *Pseudomonas aeruginosa* ATCC 27853, and *Candida albicans* ATCC 10231. To assess the impact of the prepared surface-coated materials on biofilm formation, the materials were sterilized by exposing them to ultraviolet radiation for 20 min on each side. The UVC light operated within a wavelength range of 200–280 nm, emitted from a low-pressure mercury vapor lamp at 254 nm. Each sterilized fragment was then placed individually in a well of a sterile 6-well plate. Following this, 2 mL of liquid medium (nutrient broth composed of 5 g/L peptone, 3 g/L beef extract, and 5 g/L sodium chloride) was added to each well and inoculated along with 50 μL of microbial suspension with a density of 0.5 McFarland for *S. aureus*, *P. aeruginosa*, and *C. albicans* strains. Each bacterial species was tested in three replicates (n = 3). The prepared 6-well plates containing samples were incubated at 37 °C for 24 h.

After incubation time, the materials were washed with artificial physiological solution (AFS), and the medium was replaced to facilitate further biofilm development. The plates were incubated for an additional 24 h for a mature biofilm to develop. At the end of this period, the samples with biofilm growth were washed with AFS and transferred to sterile tubes containing 1 mL of AFS. The tubes were then vigorously vortexed for 30 s to detach the cells from the biofilm. The resulting microbial cell suspension was diluted, and various dilutions were plated on a solidified culture medium (nutrient agar) to determine and quantify the number of colony-forming units (CFU/mL).

All quantitative in vitro experiments were conducted in triplicate (n = 3) to ensure the reproducibility and reliability of the results. Data were analyzed for statistical significance using GraphPad Prism software (V9), with results presented as mean ± standard deviation (SD). Statistical comparisons between groups were performed using one-way analysis of variance (ANOVA). A significance level of *p* ≤ 0.05 was considered to indicate statistically significant differences.

#### 2.3.6. In Vitro Cell-Based Assays—Biocompatibility and Oxidative Stress Production Assessment

Human preosteoblasts (hFOB 1.19 cell line) were employed as cell culture model to evaluate the biocompatibility of the tested materials. Cells were cultured following the ATCC-recommended protocols throughout the experiments. Prior to cell seeding, the samples were sterilized by UV exposure and seeded with hFOB 1.19 cells at an initial density of 2 × 10^4^ cells/cm^2^. After 30 min, materials were immersed in complete culture medium and further incubated for 72 h at 34 °C in a humidified atmosphere containing 5% CO_2_. Cell health was assessed at 24 and 72 h using the following assays:

The MTT assay was used to investigate cell viability and proliferation potential. Briefly, the culture medium was discarded and replaced with fresh MTT solution ((3-[4,5-dimethylthiazol-2-yl]-2,5 diphenyl tetrazolium bromide), Sigma Aldrich, Merck Group, Darmstadt, Germany) at the final concentration of 1 mg/mL. After 4 h of incubation under standard culture conditions, the MTT solution was removed, and the formazan crystals formed were dissolved in isopropanol. The resulting solution’s optical density (OD) was measured at 550 nm using a FlexStation III multimodal reader (Molecular Devices, San Jose, CA, USA).

To evaluate the cytotoxic potential of the materials, an LDH assay was performed. Harvested cell culture medium samples were mixed with the reagents provided in the In Vitro Toxicology TOX-7 Assay Kit, (Sigma Aldrich), following the manufacturer’s instructions. After a 30 min incubation, the reaction was terminated with HCl, and the OD of the samples was measured at 490 nm using the FlexStation III reader.

NO release was quantified using the Griess Reagent System (Promega, Madison, WI, USA). Harvested cell culture medium samples were mixed with Sulfanilamide and NED solutions provided in the kit, respecting the incubation times mentioned. After incubation, the absorbance of the samples was measured at 530 nm at the FlexStation III reader. Following the protocol presented in the kit, the nitrite standard reference curve was also prepared to aid interpreting the results.

## 3. Results

### 3.1. Powder-Type Samples Results—ZnO and ZnO-Ag

#### 3.1.1. X-Ray Diffraction (XRD)

**Figure 1:**
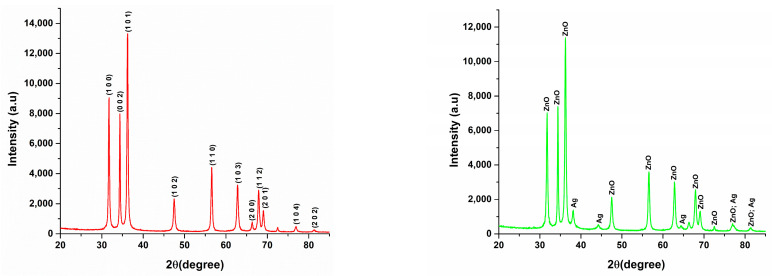
X-ray diffractograms recorded for ZnO (**left**) and ZnO-Ag (**right**) samples.

X-ray diffraction analysis offers valuable insights into the crystalline phases present in the synthesized samples, which are essential for understanding the functionality of the materials. Additionally, the diffractogram results highlight the structural differences between the two samples, allowing for an evaluation of the effects following the incorporation of silver into the ZnO-Ag composite. Both recorded diffractograms are represented in Figure 1.

The analysis of data obtained from X-ray diffraction reveals that the diffractogram for the ZnO sample aligns with the reference file coded 01-080-7099 from the PDF-ICDD database, which is specific to zinc oxide and identifies it as the sole crystalline phase present. The crystallographic structure of the sample corresponds to a hexagonal system, supported by the presence of Miller indices ((100), (002), (101), (102), (110), (103), (200), (112), (201)), which further corroborates this characterization.

In contrast, the diffractogram of the ZnO-Ag composite sample distinctly shows the diffraction peaks attributable to both zinc oxide and silver. By comparing this composite with the ZnO control sample, we can confirm the incorporation of silver into the zinc oxide structure. While the peaks associated with silver display lower intensities compared to those of ZnO—reflecting the lower concentration of silver—zinc oxide remains the predominant component in the composite. Notably, the intensities of the zinc oxide peaks also diminish, reinforcing the conclusion that silver has been successfully integrated into the antimicrobial composite. The identified database references include 01-080-7099 for zinc oxide and 04-003-7118 for silver, with corresponding Miller indices of silver being ((111), (200), (220), (311), (222)).

#### 3.1.2. Fourier Transform Infrared Spectroscopy (FT-IR)

**Figure 2:**
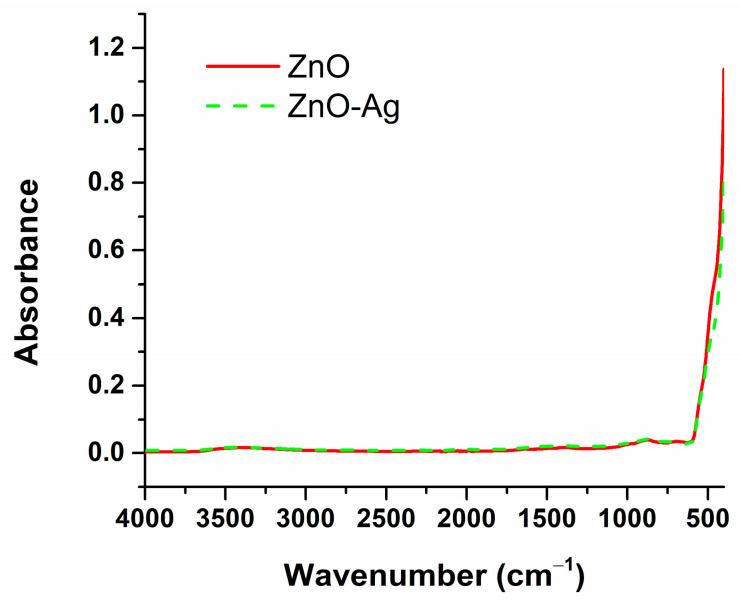
FTIR spectra obtained for ZnO and ZnO-Ag.

The samples underwent FT-IR analysis to further confirm the synthesis of zinc oxide nanoparticles and their corresponding composite nanoparticles with silver. Figure 2 illustrates the FT-IR spectra obtained for both powder samples.

The only vibrational band observed for both samples is found in the range of 400 to 500 cm^™1^. This band is attributed to the metal–oxygen functional group and highlights the stretching vibration of the zinc-oxygen bond. Additionally, the incorporation of silver into the zinc oxide structure results in a slight shift in this vibrational band. The intensity of the absorbance also changes, showing a decrease for the ZnO-Ag composite sample compared to the ZnO control sample. This alteration further confirms the successful formation of the composite material.

#### 3.1.3. Scanning Electron Microscopy (SEM)

**Figure 3:**
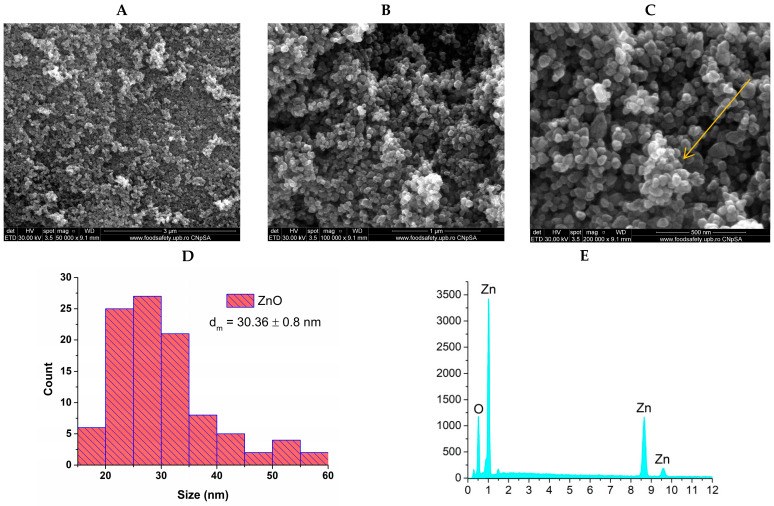
Scanning electron microscopy (SEM) micrographs at (**A**) 50,000×, (**B**) 100,000×, and (**C**) 200,000× magnifications, (**D**) particle size distribution, and (**E**) energy dispersive spectroscopy results for ZnO nanoparticles. The SEM micrographs of the ZnO sample, taken at three different magnifications (50,000, 100,000, and 200,000), confirm the formation of quasi-spherical nanoparticles, each exhibiting a consistent individual morphology and size homogeneity. The parameters of the hydrothermal synthesis process were carefully controlled to avoid accelerating the growth of the zinc oxide nanoparticles, resulting in particles with an average size of 30.36 ± 0.8 nm. Although the nanoparticles are small and exhibit a tendency for agglomeration (yellow arrow), they consistently display distinct edges and morphologies. Additionally, the EDS analysis confirms the presence of zinc (Zn) and oxygen (O) in the sample, corroborating previously obtained results.

**Figure 4:**
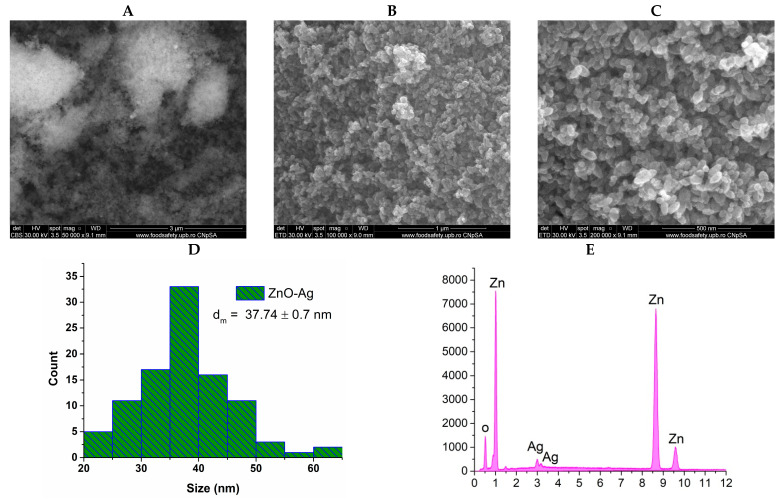
Scanning electron microscopy micrographs at (**A**) 50,000×, (**B**) 100,000×, and (**C**) 200,000× magnifications, (**D**) particle size distribution, and (**E**) energy dispersive spectroscopy results for ZnO-Ag nanoparticles.

Given that the physicochemical properties, particularly the morphology and size of the nanoparticles, significantly impact their antimicrobial activity, SEM analysis was performed. Figure 3 and Figure 4 provide essential information regarding the morphology and size of the nanoparticles.

The SEM results obtained for the composite sample of ZnO-Ag nanoparticles are presented below.

The scanning electron microscopy images of the ZnO-Ag composite sample reveal subtle differences compared to the ZnO sample, notably highlighting smaller particles that are homogenously distributed throughout the sample, which can be attributed to silver nanoparticles. The micrographs were captured at the same three magnifications as the control sample. In the image taken at 50,000× magnification, the backscattered electron mode was employed, revealing two areas with varying light intensities. The brighter area corresponds to silver, indicating its higher atomic number and greater weight relative to zinc. While the addition of silver does not alter the overall morphology of the particles, it does result in a slightly increased average size, measured at 37.74 ± 0.7 nm. Additionally, the presence of silver in the composite sample is confirmed by the identification of the Ag element in the EDS spectrum.

### 3.2. Coating-Type Samples Results—PMMA, PMMA ZnO, PMMA ZnO-Ag

#### 3.2.1. Infrared Microscopy (IRM)

**Figure 5:**
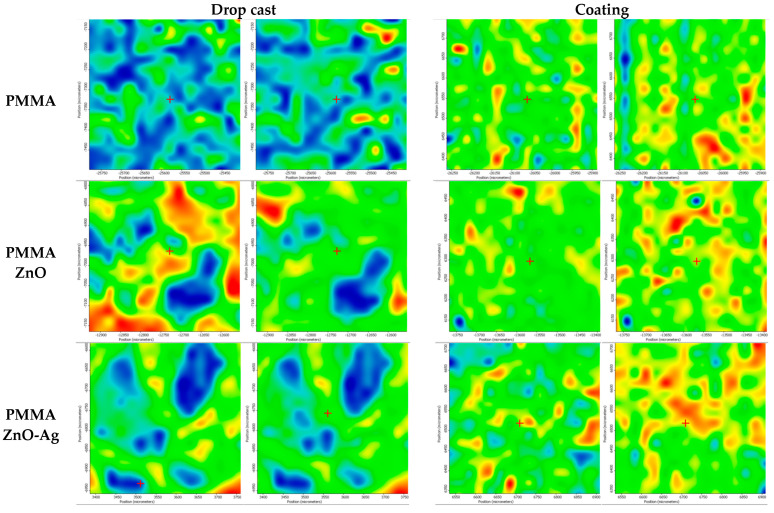
Second derivate IR mappings of PMMA, PMMA ZnO, PMMA ZnO-Ag drop cast and PMMA, PMMA ZnO, and PMMA ZnO-Ag coatings.

**Figure 6:**
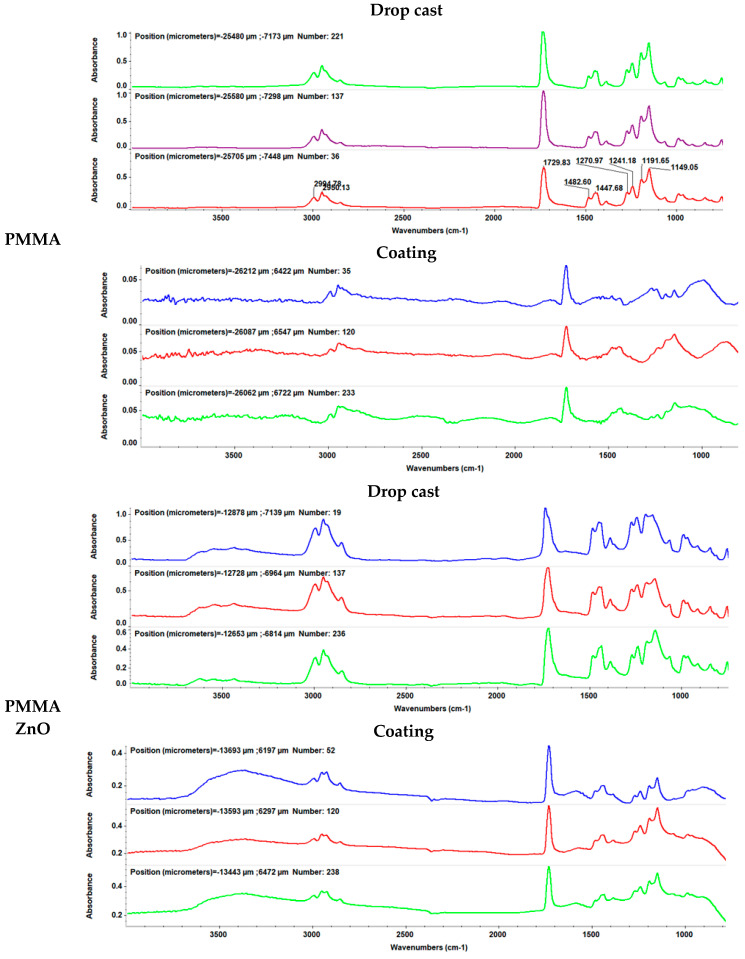

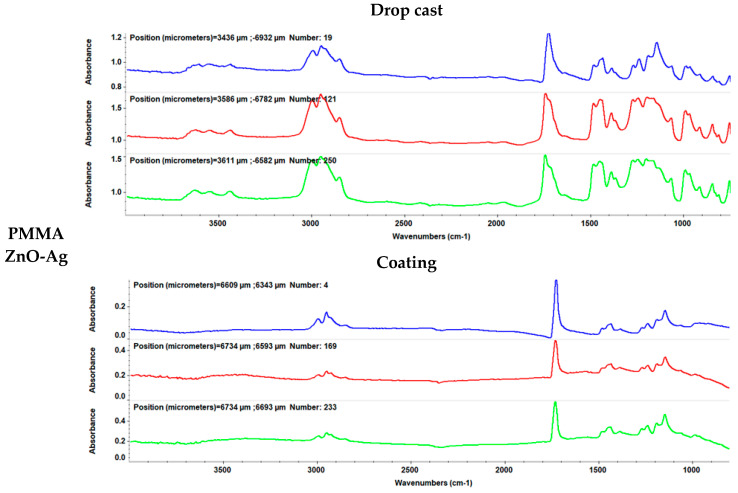
Second derivate IR spectra of PMMA, PMMA ZnO, PMMA ZnO-Ag drop casts and PMMA, PMMA ZnO, and PMMA ZnO-Ag coatings.

**Figure 7:**
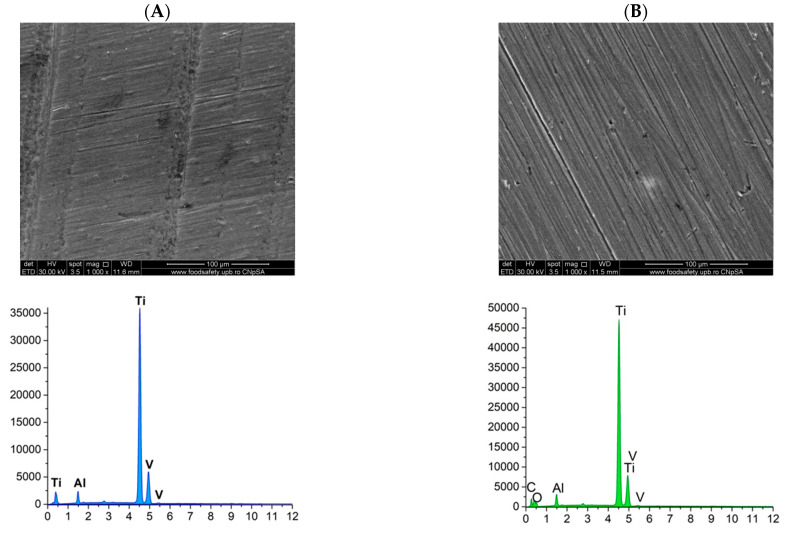

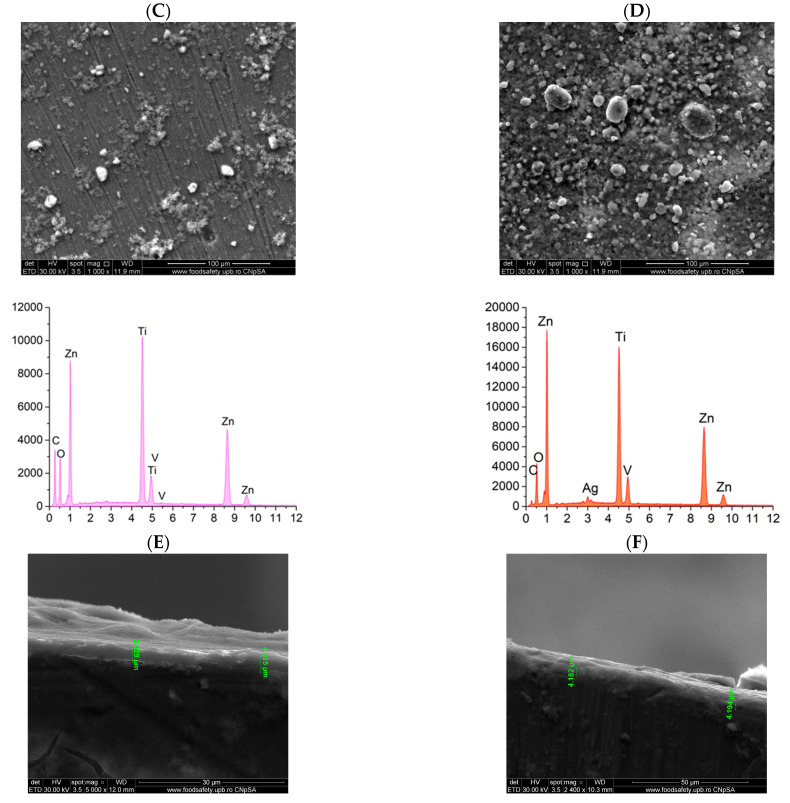
SEM micrographs and EDS spectra for Ti6Al4V uncoated substrate (**A**), PMMA coating (**B**), PMMA ZnO coating (**C**), PMMA ZnO-Ag coating (**D**), PMMA ZnO cross-section (**E**), and PMMA ZnO-Ag (**F**).

Following the experiment involving the deposition of films based on PMMA enhanced with ZnO and ZnO-Ag nanoparticles, the samples underwent a series of physical and chemical analyses to determine their properties. Given the intended application, the first analysis performed was infrared microscopy, which provided insights into the uniformity of the deposited films and facilitated the identification of the functional groups present in the materials. This information is crucial for assessing the quality of the coated samples. Figure 5, Figure 6 and Figure 7 present the FTIR microscopy results for PMMA, PMMA-ZnO, and PMMA-ZnO-Ag coatings applied to titanium alloys.

The maps generated from the analysis of all three samples—both the drop cast and PMMA-based coatings containing antimicrobial nanoparticles—exhibit varying colors, which reflect the degree and uniformity of the coatings. In the case of the drop cast samples, extensive blue regions indicate a low level of coverage. In contrast, the spin-coated samples display distinct color differences among the various samples. The PMMA control coating ranges from green to yellow, with several orange-red patches. The sample with ZnO nanoparticles shows notable differences from the control, featuring several red areas. However, the PMMA ZnO-Ag sample demonstrates superior homogeneity in its coating based on the map color, indicating enhanced integrity on the titanium alloy substrate.

Additionally, the infrared (IR) maps are correlated with the obtained IR spectra, which highlight the characteristic functional groups of the PMMA matrix. Specifically, absorption peaks at 2994 cm^™1^ and 2950 cm^™1^ are attributed to the C-H bonds associated with the stretching vibrations of CH_3_ and CH_2_ groups, respectively. Furthermore, the band observed at 1729 cm^™1^ corresponds to the acrylate carboxyl group, while the stretching vibration at 1149 cm^™1^ is associated with the C-O-C bond.

#### 3.2.2. Scanning Electron Microscopy (SEM) and Energy Dispersive X-Ray Spectroscopy (EDS) 

The PMMA-based coatings on the titanium alloy substrate were analyzed using scanning electron microscopy (SEM) to investigate the distribution of the coating on the surface and to identify the elemental composition through energy-dispersive spectroscopy (EDS) (Figure 7).

The SEM image of the titanium alloy substrate reveals noticeable surface roughness, and the elements identified through EDS analysis confirm the characteristic elemental composition of the alloy. The PMMA-coated sample displays a distinct change in surface characteristics compared to the uncoated sample, featuring a uniformly distributed and continuous layer. In the case of the PMMA-ZnO sample, numerous homogeneous nanoparticles can be observed embedded within the polymer coating, although there is a tendency for these nanoparticles to agglomerate. Conversely, the PMMA-based coating with ZnO-Ag composite nanoparticles exhibits significant changes compared to the other samples, as it contains an increase in nanoparticles presence, with nanoparticles being more uniformly distributed throughout the polymer matrix, enhancing the continuity of the deposited layers. The SEM cross-section images (Figure 7E,F) reveal the thickness variation within the samples, with measured thicknesses ranging from 2 to 4 µm for PMMA ZnO and from 4 to 5 µm for PMMA ZnO-Ag. The EDS analysis results correlate with the elemental composition, confirming the presence of characteristic elements C and O specific to PMMA, as well as Zn and Ag in each respective sample, alongside those from the alloy. This collectively reinforces the uniformity and consistency of the distribution coating across the samples.

**Figure 8:**
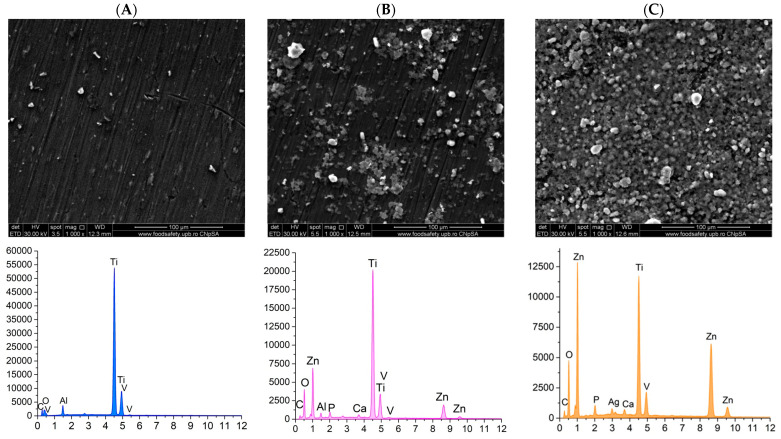
SEM micrographs and EDS spectra for the PMMA coating (**A**), PMMA ZnO coating (**B**), and PMMA ZnO-Ag coating (**C**) after 7 days of SBF immersion.

**Figure 9:**
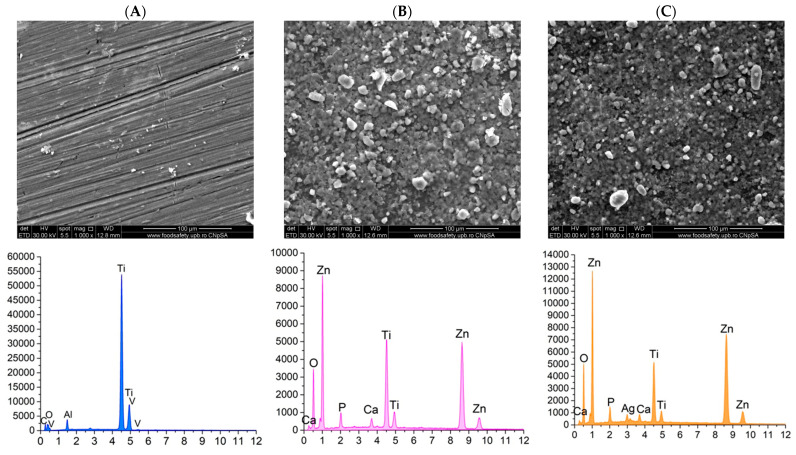
SEM micrographs and EDS spectra for PMMA coating (**A**), PMMA ZnO coating (**B**), and PMMA ZnO-Ag coating (**C**), after 14 days of SBF immersion.

**Figure 10:**
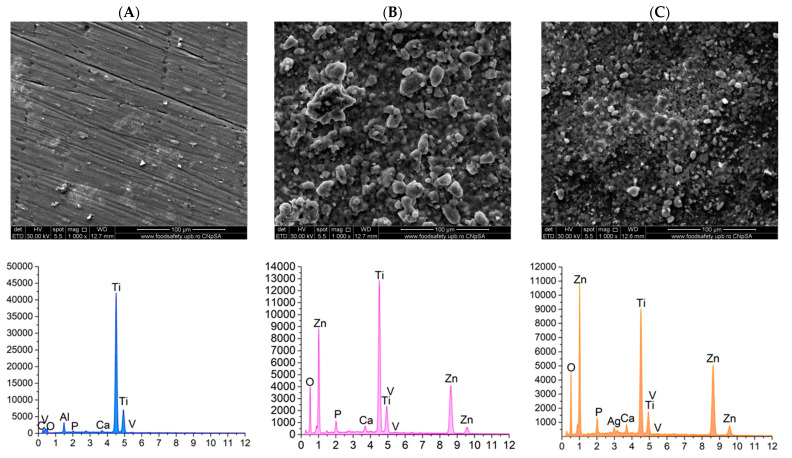
SEM micrographs and EDS spectra for PMMA coating (**A**), PMMA ZnO coating (**B**), and PMMA ZnO-Ag coating (**C**) after 21 days of SBF immersion.

The samples were subjected to SEM and EDS analysis after immersing them in simulated body fluid (SBF) prepared in accordance with Kokubo’s recipe. To simulate human body conditions, the SBF-immersed samples were maintained at 37 degrees Celsius in a water bath. At three designated time intervals—7 days, 14 days, and 21 days—the samples were removed, thoroughly washed, and analyzed to assess their interaction with SBF. The primary objective of this analysis was to observe the crystallization of calcium phosphates on the surface of the coatings, which would demonstrate the bioactivity of the materials. Figure 8, Figure 9 and Figure 10 present the SEM and EDS results corresponding to these three time intervals.

The results of the control PMMA samples after the three immersion intervals in SBF at 37 degrees Celsius reveal the presence of heterogeneous deposits on the film surface. EDS analysis confirmed that, after 7 and 14 days, the same elemental composition was observed as before immersion. However, after 21 days, new elements—specifically calcium (Ca) and phosphorus (P)—were detected. This indicates that a longer duration was necessary for the PMMA coating to facilitate the crystallization of calcium phosphate.

In contrast, the substrates coated with PMMA and enhanced with ZnO nanoparticles exhibited the formation of granulations over time. This suggests that zinc oxide nanoparticles possess superior bioactivity and promote the development of calcium phosphates when in contact with SBF. Supporting this observation, the EDS analysis revealed the presence of calcium (Ca) and phosphorus (P) alongside the characteristic elements of the sample in the spectrum. Moreover, a comparison of the results showed that, while the analysis after 7 days indicated initial findings, the intensity of the peaks associated with the elements of interest increased significantly after 14 and 21 days. This increase in peak intensity corresponds with the visual changes observed on the surface of the samples.

The samples with PMMA-based coatings incorporated with ZnO-Ag composite nanoparticles demonstrate a progressive formation of calcium phosphates, which is still time-dependent. Notably, the addition of ZnO-Ag composite nanoparticles appears to reduce the tendency for granulation-type agglomerations, resulting in a more uniform crystalline film. The bioactivity of these samples is further validated by the EDS results, which consistently confirm the presence of calcium (Ca) and phosphorus (P) across all three-time intervals examined.

#### 3.2.3. Biofilm Development

The samples developed in this study are intended to serve as covers for the abutments within a complete dental implant system. These samples aim to address the issue of infections that may arise post-implantation while also supporting the osseointegration process.

**Figure 11:**
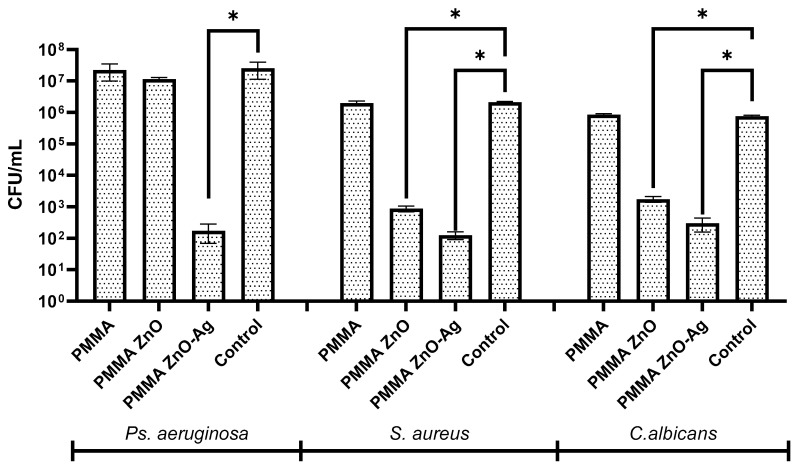
The graphic result for biofilm modulation (log10CFU/mL) of Gram-positive, Gram-negative, and fungal strains after 24 h of incubation with the coating-type samples—PMMA, PMMA ZnO, and PMMA ZnO-Ag; * *p* ≤ 0.05, one-way Anova (samples versus control).

In this context, the samples were subjected to testing focused on the dynamics of biofilm growth (initial adhesion) over a 24 h incubation period, utilizing a Gram-positive strain (*Staphylococcus aureus*), a Gram-negative strain (*Pseudomonas aeruginosa*), and a fungal strain (*Candida albicans*). The results are presented graphically in Figure 11, expressed in log10 CFU/mL. The interpretation of these results was conducted by comparing the samples to both the bacterial control and the PMMA sample.

The results reveal distinct patterns in biofilm formation across the different samples. For the PMMA control sample, the microbial counts were closely aligned with those of the untreated control, indicating no inhibitory effects. Among the tested strains, *Pseudomonas aeruginosa* exhibited the highest level of biofilm formation, with CFU/mL values exceeding 10^7^, consistent with its known resistance and biofilm-forming capability. Conversely, *Staphylococcus aureus* and *Candida albicans* demonstrated lower biofilm development, with CFU/mL values around 10^6^ for the control samples.

The PMMA ZnO samples exhibited significant antimicrobial activity, particularly against *S. aureus* and *C. albicans*, with CFU/mL values reduced to below 10^5^ and 10^4^, respectively, compared to the PMMA control. However, the inhibitory effect against *P. aeruginosa* was limited, as this strain maintained CFU/mL values of approximately 10^6^, demonstrating higher resistance to the PMMA ZnO samples. This limited effect can be attributed to the intrinsic resistance mechanisms of *P. aeruginosa*, including its outer membrane being rich in lipopolysaccharides, which act as a barrier, reducing ZnO nanoparticle penetration. Additionally, the robust extracellular polymeric substance (EPS) matrix in its biofilms further shields cells from ZnO’s antimicrobial action. These findings highlight the need for enhanced formulations to combat Gram-negative pathogens effectively.

The PMMA ZnO-Ag samples displayed the most remarkable results, with biofilm formation significantly reduced for all tested strains (*P. aeruginosa*, *S. aureus*, and *C. albicans*). The CFU/mL values for these samples were consistently reduced to approximately 10^3^, indicating a nearly complete inhibition of microbial growth. These results confirm the synergistic antimicrobial properties of zinc oxide and silver nanoparticles, effectively preventing biofilm formation within the first 24 h of exposure. The data strongly support the potential of PMMA ZnO-Ag coatings as effective antimicrobial surfaces for biomedical applications.

#### 3.2.4. In Vitro Evaluation of Materials’ Impact on Human Fibroblasts’ Health

**Figure 12:**
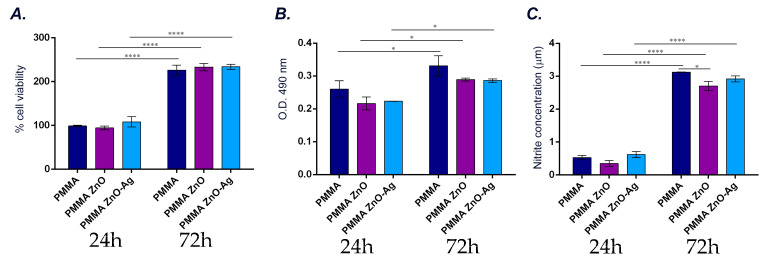
Graphical representation of (**A**) cell viability and proliferation potential, (**B**) cytotoxic potential and (**C**). nitric oxide release after 24 h and 72 h of human preosteoblasts’ interaction with PMMA, PMMA/ZnO, and PMMA/ZnO(NanoAg) coatings (statistical significance: * *p* ≤ 0.05, **** *p* ≤ 0.0001). All experiments were performed in triplicate, and the results presented represent the meaning of three independent experiments.

To investigate the effect of the tested materials on the health of hFOB 1.19 cells, a series of assays were performed to evaluate cell viability, proliferation, cytotoxicity and pro-inflammatory effects. The results of the MTT assay (Figure 12A) demonstrated that after 24 h of cell–material interaction, no significant differences in cell viability were observed between the tested coatings. This suggests that the initial interaction of human preosteoblasts with PMMA, PMMA/ZnO, or PMMA/ZnO(NanoAg) surfaces does not negatively impact their metabolic activity. Extending the interaction period to 72 h maintained a similar viability pattern, indicating that the metabolic health of hFOB 1.19 cells remained unaffected by the addition of ZnO or ZnO(NanoAg) to PMMA coatings. Notably, a more statistically significant increase in cell viability was observed at 72 h compared to 24 h across all tested materials, suggesting that these coatings support the proliferation of human preosteoblasts over time.

To further assess the potential cytotoxic effects of the coatings, LDH leakage was evaluated as a marker of cellular membrane damage (Figure 12B). The results showed that LDH levels remained consistent across all tested conditions, both at 24 and 72 h, indicating that none of the coatings induced cytotoxicity. This finding confirms that PMMA, in its simple form, and when reinforced with ZnO or ZnO(NanoAg), is non-cytotoxic to hFOB 1.19 cells.

Finally, nitric oxide (NO) release was quantified to evaluate the potential inflammatory or oxidative stress responses induced by the tested materials. NO is a key signaling molecule with elevated levels commonly associated with pro-inflammatory processes and oxidative stress. As shown in Figure 12C, NO release was comparable among PMMA, PMMA/ZnO, and PMMA/ZnO(NanoAg) coatings at both time points. This indicates that the addition of ZnO or ZnO(NanoAg) to the composite structure does not mediate pro-inflammatory effects or trigger oxidative stress in hFOB 1.19 cells.

## 4. Discussion

The field of dental medicine presents a wide array of challenges and situations akin to those found in general medicine. Infections following the implantation of restorative materials are particularly concerning, as they can lead to more severe complications. To mitigate these risks, a multidisciplinary approach that integrates chemistry and biology is essential. A promising and effective strategy involves coating abutments with a layer that possesses both antimicrobial properties and bioactivity.

Given the location and exposure of abutments within the oral environment, where food particles accumulate and bacterial populations thrive, they are especially vulnerable to biofilm formation and plaque accumulation [35,36]. When selecting materials for these protective layers, it is crucial to consider their properties to ensure suitability for dental applications. In this study, polymethyl methacrylate (PMMA) was chosen as the polymer matrix for incorporating nanoparticles and covering titanium alloy surfaces—the most commonly used material for abutment manufacturing. PMMA is favored in dentistry for its biocompatibility and ease of handling [32,37].

Additionally, the selection of nanoparticles was made based on their advantageous properties [38].

Zinc oxide nanoparticles and silver are well-studied materials known for their superior characteristics, and they can be formulated into high-performance systems with antimicrobial efficacy [39,40,41,42,43,44]. Based on our previous experience, these materials can be successfully integrated into antimicrobial composites for use in medical applications, including bioactive wound dressings [45,46] and nanostructured coatings for biomedical devices [47,48,49], holding great promise for reducing infection risks.

The properties of nanoparticles significantly affect their performance; thus, the synthesis process is of utmost importance [50,51]. In this study, we employed a hydrothermal synthesis method for these nanoparticles, allowing for precise control over temperature and pressure conditions. The synthesis resulted in two distinct powders—one for zinc oxide (ZnO) as a control and another composed of a ZnO–silver (ZnO-Ag) composite—to facilitate comparative analyses and discussions throughout the research.

The X-ray diffraction results confirm the successful synthesis of the desired phases, showcasing the high crystallinity of the samples. The presence of the Zn–O functional group, as identified through FTIR spectroscopy, supports the formation of nanoparticles. Given the variety of potential morphologies for zinc oxide nanoparticles, we optimized the synthesis parameters to yield quasi-spherical particles with nanoscale dimensions. This morphology is particularly advantageous for our intended application, as it enhances the antimicrobial activity of both types of nanoparticles. The large specific surface area increases reactivity towards bacteria, while the uniform and stable surface facilitates effective interaction with bacterial membranes, ultimately minimizing any cytotoxic effects that may arise from materials used in medical applications [52,53].

Both samples exhibited similar morphologies, reinforcing another key benefit: the incorporation of silver nanoparticles alongside zinc oxide does not alter the initial morphology of the zinc oxide particles. Additionally, the homogeneity of the samples—considering morphological, dimensional, and elemental aspects—was confirmed, further promoting their antimicrobial efficacy.

The most effective strategy for utilizing and managing nanoparticles in the development of an antimicrobial barrier on abutments is to incorporate them into a matrix suitable for deposition on the surface of the abutment. The results presented in this section pertain to PMMA-based coatings that incorporate nanoparticles synthesized via the hydrothermal method [54,55,56,57,58]. The primary objectives were to achieve a thin, uniformly distributed layer on the titanium alloy and to ensure the homogenous presence of nanoparticles throughout the polymer matrix.

To accomplish these goals, the spin coating method was selected to apply the coatings. This technique offers several advantages that align with our objectives and materials, including ease of application, precision, and optimal results. Through spin coating, the PMMA layer embedded with ZnO-Ag nanoparticles was deposited uniformly, which is crucial given the adaptive nature of bacteria; any uncovered areas could lead to adverse effects.

Additionally, each spin coating cycle generates multiple layers, with programmable parameters for rotation speed and time, allowing for precise control over each application. The uniform distribution of ZnO-Ag nanoparticles across the PMMA coating is critical for ensuring comprehensive antimicrobial efficacy. The rotational process not only aids in the distribution of nanoparticles but also enhances the adhesion of PMMA to the titanium alloy substrate [59,60,61,62]. This is particularly important given the chosen polymer’s properties. Consequently, the synthesis of the coating samples resulted in the production of three Ti6Al4V substrates: one coated with standard PMMA, one with zinc oxide nanoparticles, and a third with composite nanoparticles of zinc oxide and silver. This approach aims to maximize antimicrobial efficiency across nanoparticles-containing samples.

In exploring the theoretical aspects of selecting the spin coating method for titanium alloy substrates, we initiated our analysis by employing infrared microscopy to gather data on the distribution of the PMMA layer embedded with antimicrobial nanoparticles. The resulting maps, highlighted in distinct yellow and red hues, illustrate the uniformity of the deposition for all three materials, with particular emphasis on the PMMA ZnO-Ag composite. Furthermore, the infrared spectra reveal absorption bands that are characteristic of PMMA, thereby confirming the successful achievement of our objective: the deposition of homogeneous layers via the spin coating method on the surface of Ti6Al4V substrates [63].

Furthermore, scanning electron microscopy (SEM) conducted on both the uncoated substrate and the three coated substrates—PMMA, PMMA ZnO, and PMMA ZnO-Ag—confirms the presence, uniformity, and homogeneity of the coatings, as well as the distribution of nanoparticles on their surfaces. Concurrently, energy-dispersive X-ray spectroscopy (EDS) results indicate a consistent presence of the substrate’s characteristic elements: titanium (Ti), vanadium (V), and aluminum (Al), alongside the added zinc (Zn), oxygen (O), and silver (Ag) elements. These coated substrates were subjected to testing that simulated the human physiological environment, utilizing Kokubo’s recipe to assess the potential degradation of the coatings in contact with this medium [64]. This testing also aimed to evaluate the bioactivity of the samples by monitoring the deposition of calcium phosphates on the surfaces of the coatings [65]. Visual and elemental analyses of SEM micrographs after 7, 14, and 21 days reveal that the PMMA ZnO and PMMA ZnO-Ag samples progressively stimulate the formation of calcium phosphates over time when exposed to the simulated body fluid (SBF) solution. Notably, the uniformity of the coatings is preserved across all three time intervals, indicating strong adhesion of the layers to the titanium alloy substrate. Moreover, the presence of uniformly distributed ZnO and Ag nanoparticles, confirmed through SEM and EDS, likely underpins the enhanced antimicrobial activity observed, particularly in the PMMA ZnO-Ag coating.

After conducting the physical–chemical evaluation, it was essential to assess the primary characteristic of the synthesized materials: their antimicrobial activity. To achieve this, the samples were incubated for 24 h with a Gram-negative, Gram-positive, and fungal strains to assess their ability to modulate biofilm formation. By comparing the results with a PMMA control coating, which, according to the literature, lacks antimicrobial properties, we were able to demonstrate the efficacy of the nanoparticles incorporated into the polymer matrix. Notably, the addition of zinc oxide nanoparticles alone resulted in the complete eradication of *Staphylococcus aureus* and *Candida albicans* biofilms; however, it did not exhibit significant activity against *Pseudomonas aeruginosa*. Conversely, incorporating zinc oxide–silver composite nanoparticles yielded exceptionally effective antimicrobial activity, evidenced by the complete absence of growth or resistance among all tested bacterial and fungal strains. This suggests that the synergistic effect between zinc oxide and silver nanoparticles plays a crucial role in addressing one of the primary challenges following dental implantation, as these composite nanoparticles demonstrate effectiveness against a wide range of strains that can lead to serious infections in the oral cavity [66,67].

The results obtained in our study align with findings from another published study. Raj et al. [68] developed 2 mm-thick thin films using PMMA combined with varying percentages of commercially sourced zinc oxide powder to create formulations with potential biomedical applications. Their results demonstrated the effectiveness of these samples against *Candida albicans*. However, using commercially available zinc oxide nanoparticles resulted in elongated morphologies with an average size of 50–60 nm. By contrast, our study focused on the hydrothermal synthesis of samples in the laboratory, using parameters specifically designed to produce nanoparticles with quasi-spherical morphologies, which are directly correlated with their antimicrobial efficiency.

A study published by Zaharia et al. [69] investigates the use of acrylic beads enhanced with composite nanoparticles of ZnO and Ag, along with the incorporation of chitosan. The FTIR spectroscopy results obtained from this research are consistent with findings from studies involving PMMA coatings augmented with ZnO-Ag composite nanoparticles. Furthermore, SEM reveals a significant tendency for nanoparticle agglomeration and indicates larger particle sizes. Additionally, the assessment of antimicrobial activity is notably influenced by hybrid materials composed of PMMA, chitosan, and zinc and silver oxide nanoparticles.

The study conducted by Cierech et al. [70] aligns with the findings of our research. They developed coatings based on PMMA enhanced with zinc oxide nanoparticles synthesized using hydrothermal and solvothermal methods, with applications in dental medicine. The detailed results from their material tests closely correlate with our findings. However, there were variations in the parameters (temperature, pressure, and time) used in their hydrothermal synthesis, resulting in slightly more elongated particles. Nonetheless, they demonstrated a superior antimicrobial effect with their samples and noted that there were no significant differences in antimicrobial efficacy between the nanoparticles produced through hydrothermal and solvothermal methods.

Several studies in the literature have also considered utilizing ZnO nanoparticles and/or nanosilver in combination with other polymeric matrices for various dental applications. For instance, Toledano et al. [71] have reviewed the development of different polymeric zinc-doped nanoparticles for dentin remineralization, highlighting these nanomaterials’ antibacterial and antibiofilm potential. Additionally, Lee and colleagues [72] have recently proposed the integration of tetrapod-shaped ZnO whiskers into dual-cure dental resins, achieving potent antibacterial activity against *S. mutans*. On a different note, Garibay-Alvarado et al. [73] reported on including silver and hydroxyapatite nanoparticles in resin spheres. The developed composites exhibited bactericidal properties even at low silver concentrations, reducing oral cavity susceptibility to bacterial colonization with strains like *Ps. aeruginosa*, *S. aureus,* and *S. mutans.*

Despite being promising against tested microbial strains, the herein-described PMMA-based coatings enriched with ZnO and ZnO-Ag nanoparticles require more in-depth studies to comprehensively demonstrate their potential for dental abutments. Namely, several limitations must be addressed to determine the practical suitability of these nanostructured composites and decide upon their translation into clinical settings. First, future studies should explore the performance of these coatings in simulated oral environments, incorporating factors such as saliva, varying pH, and microbial diversity to evaluate their real-world applicability. Particularly, the duration of the antimicrobial capacity should be determined and explored against multistrain biofilms. Long-term investigations are also critical to assess the durability of the coatings under mechanical stress and their resistance to wear, as these factors are essential for dental applications. Moreover, assessing the biocompatibility of the coatings, especially the cytotoxicity of ZnO and Ag nanoparticles on surrounding tissues, is vital to ensure patient safety.

Moreover, assessing the biocompatibility of the coatings, especially the cytotoxicity of ZnO and Ag nanoparticles on surrounding tissues, is vital to ensure patient safety for prospective use in biomedical applications. For this purpose, the impact of PMMA-based coatings on human preosteoblasts was assessed and the in vitro results revealed excellent biocompatibility for all tested samples. This was observed both for PMMA in its unmodified form and for the coatings reinforced with ZnO and ZnO(NanoAg). The materials supported cell viability and proliferation without exhibiting cytotoxic or pro-inflammatory effects, highlighting their potential as promising candidates for biomedical use. The obtained results are consistent with existing literature emphasizing the biocompatibility of PMMA-based material, particularly for applications in bone regeneration and dental implants. PMMA is widely recognized as a versatile and non-cytotoxic material, frequently used in orthopedic and dental applications due to its mechanical stability and ease of processing [74,75]. The incorporation of functional additives such as ZnO and ZnO(NanoAg) into PMMA matrices has been explored to enhance antimicrobial properties and mechanical performance. A key challenge in incorporating ZnO and ZnO(NanoAg) lies in identifying the optimal concentration of these additives. The goal is to avoid cytotoxic effects while ensuring that the additives effectively deliver their intended biological benefits, such as antimicrobial activity [76,77,78]. The results of this study validate the proposed strategy, as the PMMA/ZnO(NanoAg) coatings exhibited excellent biocompatibility while also mediating antimicrobial effects, demonstrating their suitability for the intended biomedical applications.

## 5. Conclusions

This study focused on developing and testing a PMMA-based material enriched with ZnO-Ag composite nanoparticles, designed as a coating for dental abutments that are particularly susceptible to infections. The precision in hydrothermal synthesis parameters enabled the successful production of zinc oxide and zinc–silver oxide nanoparticles, characterized by a uniform quasi-spherical morphology with average particle sizes ranging from 30 to 37 nm. The analysis confirmed the presence of distinct crystallinity, relevant phases, and key functional groups. The spin coating technique was selected to apply the antimicrobial nanoparticle-rich PMMA coatings. Successful coating applications were evidenced by infrared microscopy and scanning electron microscopy results. Elemental analysis further verified the presence and homogeneous distribution of ZnO and ZnO-Ag nanoparticles within the PMMA matrix. Moreover, the evaluation of antimicrobial activity demonstrated the coatings’ enhanced performance, showing significant antimicrobial efficiency against both Gram-positive and Gram-negative bacteria and antifungal activity against Candida albicans. These findings indicate that the developed coating materials play a crucial role in inhibiting biofilm formation, thereby enhancing the antimicrobial properties of dental abutment surfaces and alleviating concerns regarding implant functionality. These findings indicate that the developed coating materials play a crucial role in inhibiting biofilm formation, thereby enhancing the antimicrobial properties of dental abutment surfaces and alleviating concerns regarding implant functionality. In vitro cell-coating material interaction studies revealed that the coatings support human preosteoblasts’ viability and proliferation without inducing cytotoxic or pro-inflammatory effects, validating their potential for safe use in biomedical applications.

## Data Availability

The original contributions presented in this study are included in the article. Further inquiries can be directed to the corresponding author.
